# Gender Differences in Cardiovascular Risk Factors, Clinical Presentation, and Outcome of Patients Admitted with a Hypertensive Crisis at the Buea Regional Hospital, Cameroon

**DOI:** 10.1155/2022/3062526

**Published:** 2022-06-28

**Authors:** Clovis Nkoke, Ahmadou Musa Jingi, Jean Jacques Noubiap, Denis Teuwafeu, Cyrille Nkouonlack, Ronald Gobina, Siddikatou Djibrilla, Ali Abas, Anastase Dzudie

**Affiliations:** ^1^Faculty of Health Sciences, University of Buea, Buea, Cameroon; ^2^Clinical Research Education, Networking and Consultancy, Douala, Cameroon; ^3^Faculty of Health Sciences, The University of Bamenda, Bamenda, Cameroon; ^4^Centre for Heart Rhythm Disorders, South Australian Health and Medical Research Institute, University of Adelaide and Royal Adelaide Hospital, Adelaide, Australia; ^5^Faculty of Medicine and Biomedical Sciences, University of Ngaoundere, Yaounde, Cameroon; ^6^Faculty of Medicine and Biomedical Sciences, University of Yaounde 1, Ngoa Ekélé, Cameroon

## Abstract

**Background:**

Several recent studies have shown differences in the risk profile and outcome of cardiovascular diseases between men and women, with a dearth of data from African populations. This study aimed to examine gender differences in a group of patients from Cameroon hospitalized with a hypertensive crisis.

**Methods:**

We conducted a cross-sectional study from June 2018 until June 2019. The criteria to define a hypertensive crisis (HC) were systolic and/or diastolic blood pressure should be ≥180/110 mmHg. We compared the clinical presentation and outcome of males versus females.

**Results:**

Out of the 1536 patients admitted, 95 (6.2%) had an HC. There were 49 (51.6%) men. There was no significant age difference between men and women (52.7 years vs. 49.3 years, *p* = 0.28). Alcohol consumption (*p* < 0.0001), previous stroke (*p* = 0.04), and smoking (*p* = 0.03) were significantly higher in men compared to women. Men had a higher proportion of psychomotor agitation (*p* = 0.05). There was an equal proportion of men and women with hypertensive emergencies. Although acute left ventricular failure was most frequent in women (46.4% vs 42.9%), cerebral infarction (14.3% vs 17.9%), and acute coronary syndrome (0% vs 7.1%) were higher in men, the differences were not statistically significant (all *p* > 0.05). Case fatality was also higher in men compared to women but the difference was not statistically significant.

**Conclusions:**

Men admitted for an HC had a significantly higher cardiovascular risk burden and higher psychomotor agitation. However, there were no significant differences in the types of hypertensive emergencies and outcomes between men and women.

## 1. Introduction

Cardiovascular diseases (CVD) are the leading cause of death globally for both men and women [1]. Cardiovascular disease includes a broad and diverse range of subtypes like myocardial infarction, cerebrovascular disease, hypertension, and heart failure. The burden of CVD can be reduced by keeping blood pressure, cholesterol, and glucose at healthy levels; avoiding tobacco; and maintaining a healthy weight [2]. Studies have shown gender differences in the occurrence of the various forms of CVD. Men have a higher risk of coronary heart disease than women, especially at younger ages [3]. Women, on the other hand, have a similar or greater risk of developing stroke and heart failure [4–6].

A hypertensive crisis (HC) is one of the major acute complications of hypertension, resulting in an emergency admission to the hospital. It is estimated that 1 to 2% of hypertensive patients will develop a hypertensive crisis as a complication of untreated or poorly controlled hypertension [7]. Gender differences in the incidence and severity of hypertension are well established whereby males have a higher incidence of hypertension compared to females of the same age until the sixth decade of life [8, 9]. Previous studies found a higher prevalence of hypertensive crises in women [10–12]. A few studies have compared the differences in hypertensive crises between men and women in Cameroon.

This study aimed to assess the gender differences in risk factors, clinical presentation, and outcome in patients hospitalized with a hypertensive crisis in the Buea Regional Hospital.

## 2. Methods

### 2.1. Study Design and Setting

This was a cross-sectional study conducted in the Department of Internal Medicine of the Buea Regional Hospital, between June 2018 and June 2019. This is a secondary-level hospital that serves as one of the two main referral centers in the South West region and is the main teaching hospital of the University of Buea. It has a capacity of about 111 beds and a catchment population of about 200,000 inhabitants. Buea is a semiurban setting, and the main economic activity is agriculture.

### 2.2. Study Population

This is a subanalysis of previously published data. The study methodology has been published previously [13]. The criteria to define hypertensive crisis were systolic and/or diastolic blood pressure ≥180/110 mmHg. Hypertensive urgency was defined as a marked elevation in blood pressure (systolic ≥180 or diastolic ≥110 mmHg) with no evidence of acute end-organ damage. Hypertensive emergency was defined as a blood pressure ≥180 for the systolic or ≥110 mmHg for the diastolic, with evidence of acute end-organ damage including hypertensive encephalopathy, cerebral infarction, intracerebral hemorrhage, retinopathy, acute left ventricular failure, acute coronary syndrome, acute renal injury, and aortic dissection [4]. Hypertensive encephalopathy was defined as severe BP elevation associated with lethargy, seizures, cortical blindness, and coma in the absence of other explanations. A neurologic deficit was defined as a focal motor or sensory deficit (ex: hemiparesis/hemiplegia, sensory loss). In the absence of evidence for acute end-organ damage, all other hypertensive crises were considered by exclusion to be hypertensive urgencies.

### 2.3. Data Collection

All patients underwent a complete clinical examination. We collected data on age, sex, previous history of hypertension and treatment, cardiovascular risk factors and comorbidities, any relevant symptoms, systolic and diastolic blood pressure, clinical features of acute organ damage, electrocardiogram, serum creatinine, length of hospital stay, and in-hospital mortality. Cardiac troponin for myocardial ischemia or infarction, computed tomography was done when indicated. The blood pressure was measured with an automated BP machine by experienced state registered nurses following standard procedures. The blood pressure measurement machine used was the OMRON® M3 Intellisense. We considered the average of two consecutive blood pressure measurements.

### 2.4. Ethical Consideration

The study was approved by the institutional review board of the Buea Regional hospital, reference number: NT/MPH/SWRDPH/BRH/IRB 206.

### 2.5. Data Analysis

The data were analyzed using SPSS 20 for Windows. Data were presented as the mean with standard deviation (SD) or median (interquartile ranges) for continuous variables, and percentages (%) for categorical variables. The difference in frequencies between men and women with hypertensive emergencies and hypertensive urgencies was analyzed using the chi-square or Fisher's exact test. A student's *t*-test or Mann–Whitney *U* test was used to compare continuous variables between the two groups of men and women. We used logistic regression to assess factors associated with death. A *p* value less than 0.05 was considered significant.

## 3. Results

### 3.1. Sociodemographic Characteristics, Risk Factors, and Comorbidities

Out of the 1536 patients admitted to the medical unit, 95 (6.2%) were diagnosed with a hypertensive crisis. There were 49 (51.6%, 95% CI: 41.1–62) males and 46 (48.4%, 95%CI: 38–58.9) females. The sociodemographic characteristics risk factors and comorbidities of the affected patients are shown in Table 1. The mean age was 51.1 ± 14.9 years and ranged from 14 to 86 years. There was no significant age difference between men and women (52.9 ± 13.2 years vs 49.3 ± 16.6 years, *p*=0.28). A history of previously diagnosed hypertension was reported by 72 (75.8%, 95% CI: 65.9–84) patients. The proportions of alcohol consumption (*p* < 0.0001), previous stroke (*p*=0.04), and smoking (*p*=0.03) were significantly higher in men compared to women.

### 3.2. Clinical Characteristics, Types of Hypertensive Emergencies, and Outcomes

Signs and symptoms most frequently found in the cohort overall were headache (34.7%), dyspnea (34.7%), neurological deficit (23.2%), and chest pain (17.9%). Compared to women, men had a significantly higher proportion of psychomotor agitation (*p*=0.05). There was no statistically significant difference in mean systolic and diastolic blood pressures between men and women (Table 2). A hypertensive emergency (HE) was seen in 56 (59%, 95% CI: 48.4–68.9) patients. There was no significant difference in HE between genders (male vs female: 57.1% vs 60.9%, *p*=0.712). The most frequent forms of HE were acute left ventricular failure with pulmonary edema (44.6%), intracerebral hemorrhage (21.4%), and cerebral infarction (16.1%). The proportion of acute left ventricular failure was higher in women, while the proportion of cerebral infarction was higher in men. The prevalence of acute coronary syndrome was 3.6% with all cases occurring in men. However, these differences were not statistically significant (Table 3). Overall, brain involvement was seen in 26 (27.4%, 95% CI: 18.7–37.5) patients, heart involvement in 27 (28.4%, 95% CI: 19.6–38.6) patients, and kidney involvement in 4 (4.2%, 95% CI: 1.2–10.4) patients. Two organ involvement was seen in 1 patient. There was no gender difference in target organ involvement (all *p* > 0.5). The in-hospital case-fatality was seen in 6.3% (95% CI: 2.4–13.2) of patients. The mean length of hospital stay was 8.4 ± 4.6 days (range: 1 to 21 days). There was no significant difference in the mean length of hospital stay and mortality between men and women. Those with a hypertensive emergency were more likely to die compared with those with hypertensive urgency (OR: 3.7, 95% CI: 0.4–33.2). In bivariate analysis, patients with cerebral infarction were more likely to die (OR: 5.9, 95% CI: 0.9–37.8) Table 4. No factor was independently associated with death in multivariate analyses.

## 4. Discussion

This study aimed to examine the gender differences in risk factors, clinical presentation, and outcome in patients hospitalized with a hypertensive crisis in a hospital in a semiurban setting in Cameroon. Our results show that men admitted for a hypertensive crisis had a significantly higher proportion of alcohol consumption (*p* < 0.0001), previous history of stroke (*p*=0.04), and smoking (*p*=0.03) compared to women, and a higher proportion of psychomotor agitation. There was almost an equal proportion of men and women with HC. Also, there were no significant differences in different types of hypertensive emergencies and outcomes between men and women.

Cardiovascular disease is the leading cause of death globally in both men and women [14]. One presentation or even consequence of inadequate blood pressure control in patients with hypertension is a hypertensive crisis. Considerable gender differences exist in the occurrence of various manifestations of cardiovascular diseases [3–6]. In this study, men were older than women (52.7 years vs 49.3 years, *p* = 0.28) but the age difference was not statistically significant. This is contrary to reports in the literature where men generally develop cardiovascular disease at a younger age compared to women [3, 15]. Cardiovascular disease develops 7 to 10 years later in women than in men [3, 15]. In a similar study on hypertensive crisis in India, men were younger compared to women, which was different from the results in our study [16]. Also, there was an equal proportion of men and women with hypertensive emergencies in our study (50% in each gender). This result was different from findings in India where 60% of the patients with a hypertensive emergency were males [16]. Most previously published studies found a higher prevalence of hypertensive crisis in women [10–12]. But a recent systematic review showed that hypertensive emergencies were more common in men [17]. The prevalence of hypertensive crisis in women in our study may have been underestimated because we excluded patients with pre-eclampsia and eclampsia. Patients with pre-eclampsia and eclampsia who have symptoms in our hospital are treated in obstetrics.

In our study, men had a significantly higher cardiovascular risk factor burden compared to women. The prevalence of smoking, alcohol consumption, and previous strokes were significantly higher in men. Although CVD usually occurs at a younger age in men, men have a higher risk of coronary artery disease than women, while women are at a higher risk of stroke which usually occurs at an older age [18, 19]. Smoking is a leading modifiable cause of cardiovascular disease morbidity and mortality [20].In our study, the proportion of smokers was significantly higher in men. Significant gender differences in smoking have been reported. Generally, the age-standardized prevalence of smoking in men is higher than that in women, and women have a higher expectation of self-control than men [21–23]. Similar to smoking, the prevalence of alcohol consumption was higher in men compared to women. Generally, male drinkers tend to drink more often and more heavily than females do [24]. A recent systematic review on hypertensive crises showed that the risk of hypertensive crisis is higher in patients with unhealthy alcohol consumption [17]. The recent review also showed that the risk of the hypertensive crisis was higher in patients with a history of a comorbid cardiovascular condition such as stroke [17]. The proportion of strokes in men in our study was significantly higher than in our previous study.

In our study, there were no significant differences between men and women concerning the different types of hypertensive emergencies. We reported only 2 cases of acute coronary syndrome in the study, but all of these cases occurred in men. Although this number was small, it can be explained by the significantly higher proportion of smoking in men. Smoking is a major risk factor for coronary artery disease. It increases the incidence of myocardial infarction and fatal coronary artery diseases [25]. Women with clinically manifested coronary artery disease are generally older than men, with a higher expression of cardiovascular risk factors [26, 27]. But the males in our study were slightly older than the females. Also, the proportion of cerebral infarction was higher in men compared to women, though not statistically significant. The incidence of cerebral infarction is generally higher in men compared to women [28]. Regarding the outcome, there were no statistically significant differences in mortality and length of hospital stay between males and females even though these were higher in males. Generally, cardiovascular disease mortality is higher in women compared to men [29].

## 5. Limitations

Our study is limited by its small size. This might have prevented the detection of significant differences between some variables in men and women. Furthermore, our study was conducted only in the referral hospital for the region in the urban area. Patients from remote or rural areas might have been underrepresented. We also excluded patients with pre-eclampsia and eclampsia. Despite these limitations, our study is one of the few studies in our setting which has assessed gender differences in hypertensive crisis and served as the basis for future larger studies.

## 6. Conclusion

Males admitted for a hypertensive crisis had a significantly higher cardiovascular risk burden and higher psychomotor agitation. However, there was no significant difference in the types of hypertensive emergencies and outcomes between males and females. These findings need to be confirmed by larger studies.

## Figures and Tables

**Table 1:** Sociodemographic characteristics, risk factors, and comorbidities.

Variable	Male (*n* = 49)	Female (*n* = 46)	Overall (*n* = 95)	*p* value
Age (years), mean (SD)	52.7 ± 13.2	49.3 ± 16.7	51.1 ± 14.9	0.28
History of hypertension, *n* (%)	37 (75.5)	34 (73.9)	71 (74.7)	0.72
Treatment of hypertension, *n* (%)	11 (22.4)	12 (26.1)	23 (24.2)	0.82
Chronic kidney disease, *n* (%)	12 (24.5)	10 (21.7)	22 (23.2)	0.71
Diabetes, *n* (%)	5 (10.2)	8 (17.4)	13 (13.7)	0.33
Alcohol, *n* (%)	26 (53.1)	5 (10.9)	31 (32.6)	<0.0001
Smoking, *n* (%)	6 (12.2)	0	6 (6.3)	0.03
Previous stroke, *n* (%)	4 (8.2)	0	4 (4.2)	0.04
Ischemic heart disease, *n* (%)	1 (2)	0	1 (1.1)	0.33
Dyslipidemia, *n* (%)	1 (2)	0	1 (1.1)	0.33
Heart failure, *n* (%)	4 (8.2)	1 (2.2)	5 (5.3)	0.18

**Table 2:** Clinical presentation and outcome.

Variable	Male (*n* = 49)	Female (*n* = 46)	Overall (*n* = 92)	*p* value
Systolic BP (mmHg), mean ± SD	209.2 ± 22.5	202.7 ± 22.1	206.1 ± 22.3	0.16
Diastolic BP (mmHg), mean ± SD	129.0 ± 16.9	130.9 ± 18.8	129.7 ± 17.9	0.61
Initial loss of consciousness, *n* (%)	5 (10.2)	4 (8.7)	9 (9.8)	0.77
Glasgow coma score, mean ± SD	14.7 ± 1.4	14.3 ± 2.4	14.5 ± 1.9	0.33
Headache, *n* (%)	20 (40.8)	13 (28.3)	23 (24.2)	0.17
Convulsions, *n* (%)	4 (8.2)	1 (2.2)	5 (5.3)	0.18
Dyspnea, *n* (%)	15 (30.6)	18 (39.1)	33 (34.7)	0.42
Chest pain, *n* (%)	10 (20.4)	7 (15.2)	17 (17.9)	0.45
Vomiting, *n* (%)	6 (12.2)	6 (13)	12 (12.6)	0.94
Neurological deficit	14 (28.6)	8 (17.4)	22 (23.2)	0.16
Psychomotor agitation, *n* (%)	4 (8.2)	0	4 (4.2)	0.05
Creatinine (mg/l), median(IQR)	26 (13–87)	16.6 (10–82)	21 (12–82)	0.413
Hemoglobin (g/dl), mean ± SD	11.2 ± 2.6	10.6 ± 2.9	10.9 ± 2.7	0.49
Total cholesterol (g/l), mean ± SD	172 ± 48.9	158.9 ± 67.3	168.6 ± 58.0	0.52
Death, *n* (%)	4 (8.2)	2 (4.3)	6 (6.3)	0.38
Length of hospital stay (days), mean ± SD	8.9 (4.4)	7.9 (4.7)	8.4 ± 4.6	0.75

**Table 3:** Types of hypertensive emergencies.

Types of hypertensive emergencies	Male (*n* = 28)	Female (*n* = 28)	Overall (*n* = 56)	*p* value
Hypertensive encephalopathy, *n* (%)	1 (3.5)	1 (3.5)	2 (3.5)	NA
Cerebral infarction, *n* (%)	5 (17.9)	4 (14.3)	9 (16.1)	0.716
Intracerebral hemorrhage, *n* (%)	6 (21.4)	6 (21.4)	12 (21.4)	NA
Acute left ventricular failure, *n* (%)	12 (42.9)	13 (46.4)	25 (44.6)	0.794
Acute coronary syndrome, *n* (%)	2 (7.1)	0	2 (3.6)	0.155
Undetermined CVA, *n* (%)	2 (4.1)	1 (2.2)	3 (3.2)	0.687
Acute kidney injury, *n* (%)	2 (4.1)	2 (4.3)	4 (4.2)	0.971
Hypertensive retinopathy, *n* (%)	1 (2)	0	1 (1.1)	0.456

**Table 4:** Factors associated with death in the study population (*n* = 95)

Variables	Death, N (%)	OR (95% confidence interval)	*p* value
Female gender			
Yes	4 (8.7)	2.2 (0.4–12.8)	0.43
No	2 (4.1)	1	
Age > 50 years			
Yes	4 (7.8)	1.8 (0.3–10.3)	0.68
No	2 (4.6)	1	
Known hypertension			
Yes	6 (8.3)	NA	0.33
No	0 (0)	1	
Known chronic kidney disease			
Yes	0 (0)	NA	0.33
No	6 (8.3)	1	
Diabetes			
Yes	0 (0)	NA	0.59
No	6 (7.4)	1	
Previous stroke			
Yes	1 (25)	5.7 (0.5–65.5)	0.23
No	5 (5.5)	1	
Previous heart failure			
Yes	1 (20)	4.3 (0.4–45.5)	0.28
No	5 (5.6)	1	
Alcohol consumption			
Yes	1 (3.1)	0.4 (0.04–3.4)	0.66
No	5 (7.9)	1	
Tobacco consumption			
Yes	0 (0)	NA	NA
No	6 (6.8)	1	
Hypertensive emergency			
Yes	5 (8.9)	3.7 (0.4–33.2)	0.39
No	1 (2.6)	1	
Intracerebral hemorrhage			
Yes	1 (8.3)	1.4 (0.2–13.3)	0.57
No	5 (6)	1	
Cerebral infarction			
Yes	2 (22.2)	5.9 (0.9–37.8)	**0.09**
No	4 (4.7)	1	
Hypertensive encephalopathy			
Yes	0 (0)	NA	NA
No	6 (6.5)	1	
Pulmonary edema			
Yes	2 (8)	1.4 (0.3–8.4)	0.65
No	4 (5.7)	1	
Acute kidney injury			
Yes	0 (0)	NA	NA
No	6 (6.6)	1	

## Data Availability

The datasets used and/or analyzed during the current study are available from the corresponding author on reasonable request.

## Abbreviations

CVD: Cardiovascular diseases
HE: Hypertensive emergencies
HC: Hypertensive crisis.
